# Vasoconstriction with phenylephrine increases cardiac output in preload dependent patients

**DOI:** 10.1007/s10877-024-01186-7

**Published:** 2024-06-21

**Authors:** Jakob Højlund, Mirjana Cihoric, Nicolai Bang Foss

**Affiliations:** Department of Anaesthesiology, Hvidovre University Hospital, Capital Region, Denmark

**Keywords:** Preload dependency, Vasopressor, Vasopressor preload modulation, Phenylephrine, Lithium dilution cardiac output

## Abstract

General Anaesthesia (GA) is accompanied by a marked decrease in sympathetic outflow and thus loss of vasomotor control of cardiac preload. The use of vasoconstriction during GA has mainly focused on maintaining blood pressure. Phenylephrine (PE) is a pure α1-agonist without inotropic effects widely used to correct intraoperative hypotension. The potential of PE for augmenting cardiac stroke volume (SV) and -output (CO) by venous recruitment is controversial and no human studies have explored the effects of PE in preload dependent circulation using indicator dilution technique. We hypothesized that PE-infusion in patients with cardiac stroke volume limited by reduced preload would restore preload and thus augment SV and CO. 20 patients undergoing GA for gastrointestinal surgery were monitored with arterial catheter and LiDCO unity monitor. Upon stable haemodynamics after induction patients were placed in head-up tilt (HUT). All patients became preload responsive as verified by a stroke volume variation (SVV) of > 12%. PE-infusion was then started at 15-20mikrg/min and adjusted until preload was restored (SVV < 12%). Li-dilution cardiac output (CO) was initially measured after induction (baseline), again with HUT in the preload responsive phase, and finally when preload was restored with infusion of PE.At baseline SVV was 10 ± 3% (mean ± st.dev.), CI was 2,6 ± 0,4 L/min*m^2^, and SVI 43 ± 7mL/m^2^. With HUT SVV was 19 ± 4%, CI was 2,2 ± 0,4 L/min*m^2^, SVI 35 ± 7mL/m^2^. During PE-infusion SVV was reduced to 6 ± 3%, CI increased to 2,6 ± 0,5 L/min*m^2^, and SVI increased to 49 ± 11mL/m^2^. All differences *p* < 0,001. In conclusion: Infusion of phenylephrine during preload dependency increased venous return abolishing preload dependency as evaluated by SVV and increased cardiac stroke volume and -output as measured by indicator-dilution technique. (ClinicalTrials.gov NCT05193097).

## Purpose

Hypotension is very common during general anaesthesia (GA) and is associated with adverse perioperative outcomes [1]. The hypotension of anaesthesia is per tradition attributed to arteriolar dilatation leading to lowering of systemic vascular resistance [2]. However, besides the loss of afterload, hypotension can be caused by reduction of CO due to loss of cardiac contractility, decreased heart rate, and/or decreased preload due to venous dilatation.

Correction of hypotension is one of the most frequent interventions of the anaesthesiologist and the relative indications for the use of either fluids or vasoconstriction is a subject of scientific and clinical debate. The prevalent paradigm is based on correction of preload with fluids to optimize flow, and subsequent correction of afterload with vasoconstriction [3].

Intraoperative infusion of vasopressor is often performed with phenylephrine. Phenylephrine is a pure α1-agonist without inotropic effects, often described to decrease cardiac output due to increasing SVR [4]. However, growing evidence suggest that PE indeed may increase SV and thereby CO when the heart is preload dependent. Venoconstriction by PE will increase stressed volume, thus increasing venous return and CO [5]. As such, the effect of PE depends on the position of the heart on the Frank-Starling relationship [6–9]. These studies, however, have been based on methods (ultrasound flow estimation or pulse contour/power analysis) that are susceptible to a number of bias and do not perform well during pharmacological vasoconstriction [10–12]. The effect of phenylephrine on CO in preload dependent patients has not been investigated using indicator dilution technique, which is considered the gold standard.

We hypothesized that PE-infusion during preload-dependency induced by head-up tilt would increase venous return, restore preload, and thus increase cardiac stroke volume and -output.

## Methods

The study was approved by the regional ethics committee (H-21032981) and registered at ClinicalTrials.gov (NCT05193097). Processing of personal data was approved by the Danish Data Protection Agency (AHH-2016-095).

### Study population

For this prospective observational study we included 21 patients scheduled for gastrointestinal surgery under general anaesthesia at the central operating theatre at Hvidovre University Hospital. Patients with atrial fibrillation, heart failure, or cerebral vascular disease were excluded. All patients gave oral and written consent to participate. Inclusion was performed the day before surgery; and only when feasible, i.e. when both MC and JH was available on the study day. No patients retracted consent; one patient was excluded on the study day due to equipment failure.

### Study setting

All patients had an arterial catheter placed in the radial artery with a LiDCO Unity monitor (LiDCO, London, UK) connected providing continuous readings of mean arterial pressure (MAP) and heart rate (HR). For measuring CO, a lithium sensitive electrode was connected to the arterial line and a roller pump drew arterial blood at constant rate to obtain arterial lithium concentration-time curve for the monitor to calculate CO using the Stewart-Hamilton equation. Lithium 0,3 mmol was injected using a central line or a suitable peripheral vein (external jugular/basilic/brachial). For each measuring period, CO was measured twice, and the mean was used. If measurements differed more than 15% a third measurement was performed.

Induction of anaesthesia was performed in the supine position at the discretion of the investigator (JH) with Propofol (1,5-2 mg/kg), Remifentanil (6–10 μg/kg), and Succinylcholine (1 mg/kg) if RSI was indicated. Following tracheal intubation tidal volumes of 8mL/kg ideal body weight was used with frequency adjusted to normoventilation by end-tidal CO_2_. PEEP was 6 cmH_2_O. Anaesthesia was maintained with Propofol 50–75 μg/kg/min and Remifentanil 0,5 − 0,8 μg/kg/min. If MAP was below 60 prior to the baseline measurement ephedrine 10 mg was administered.

All study measurements were performed before the start of surgery. With steady readings of algorithmic CO and MAP after induction the baseline lithium dilution CO was measured as described. Then the table was tilted in moderate HUT position of 25 degrees. When a new steady state was reached lithium dilution CO was measured. Finally, with the patient still in HUT, a phenylephrine-infusion was started at a rate of 15–20 μg/min, aiming for a stroke volume variation (SVV) < 12%. Upon steady state lithium dilution CO was measured.

### Data analysis

Lithium dilution CO was registered in real time as described. MAP, HR, and SVV were exported from the LiDCO monitor and analyzed off-line using LiDCOviewPRO V1 (LiDCO, London, UK). MAP, HR, and SVV were calculated as the mean of 15s before and 15s after the lithium dilution CO measurements (where there was no signal due to the arterial cannula being used for the lithium dilution measurements). Cardiac Index (CI) was lithium dilution CO/BSA using the DuBois formula for body surface area; SVI was CI/HR.

### Statistics

The distribution of data was tested with the Kolmogorow-Smirnov test for normality and inspected graphically using qq-plots. Data are reported as mean with std. deviation. Differences between haemodynamic outcome variables were evaluated using paired T-tests. All analyses were performed using GraphPad Prism statistical software v10 (GraphPad Software, Boston, MA, USA) and a 2-sided *p*-value < 0.05 was considered statistically significant.

**Table 1:** Patient characteristics

Patients, n	20
Female, n (%)	11 (55)
Age, years ± SD	59 ± 15
Height, cm ± SD	171 ± 8
Weight, kg ± SD	82 ± 17
Body Surface Area, m^2^ ± SD	1,94 ± 0,2
ASA physical status, n (%)	
1	4 (20)
2	13 (65)
3	3 (15)
4	0 (0)
Co-morbidities, n (%)	
Hypertension	8 (40)
Smoking	3 (15)
Ischemic heart disease	2
COPD/Asthma	6
Diabetes mellitus	1
Cardiovascular Medication	
Hydrochlorthiazide	4
ACE inhibitors / AT2 blockers	6
Calcium channel blockers	3
Β-receptor antagonists	1
Loop diuretics	2
Type of surgery	
Upper GI	10
Lower GI	8
Hernia repair	2

## Results

Of the 20 patients that completed the study 11 were female, age was 59 ± 15 years (mean ± st.dev.), height 171 ± 8 cm, weight 82 ± 17 kg, and BSA (DuBois) 1,94 ± 0,2 m^2^. Patient characteristics are presented in Table 1.

Baseline CI was 2,6 ± 0,4 L/min*m^2^, MAP was 64 ± 10 mmHg, and SVV was 10 ± 3%. With HUT CI decreased by 15% to 2,2 ± 0,4 L/min*m^2^, and MAP decreased by 33% to 43 ± 8 mmHg (Table 2).

**Fig. 1:**
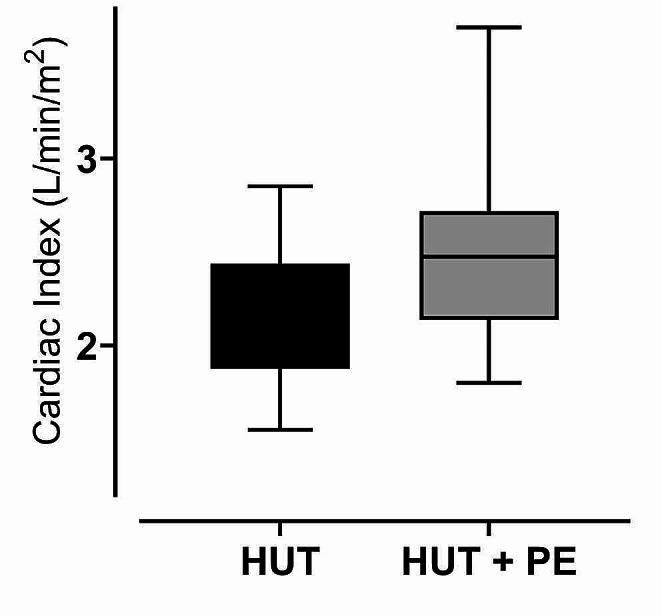
Cardiac index during head-up tilt before and after phenylephrine infusion Cardiac Index during Head-Up Tilt (HUT) and during Head-Up Tilt with Phenylephrine infusion (HUT + PE). *p* < 0,001

**Fig. 2:**
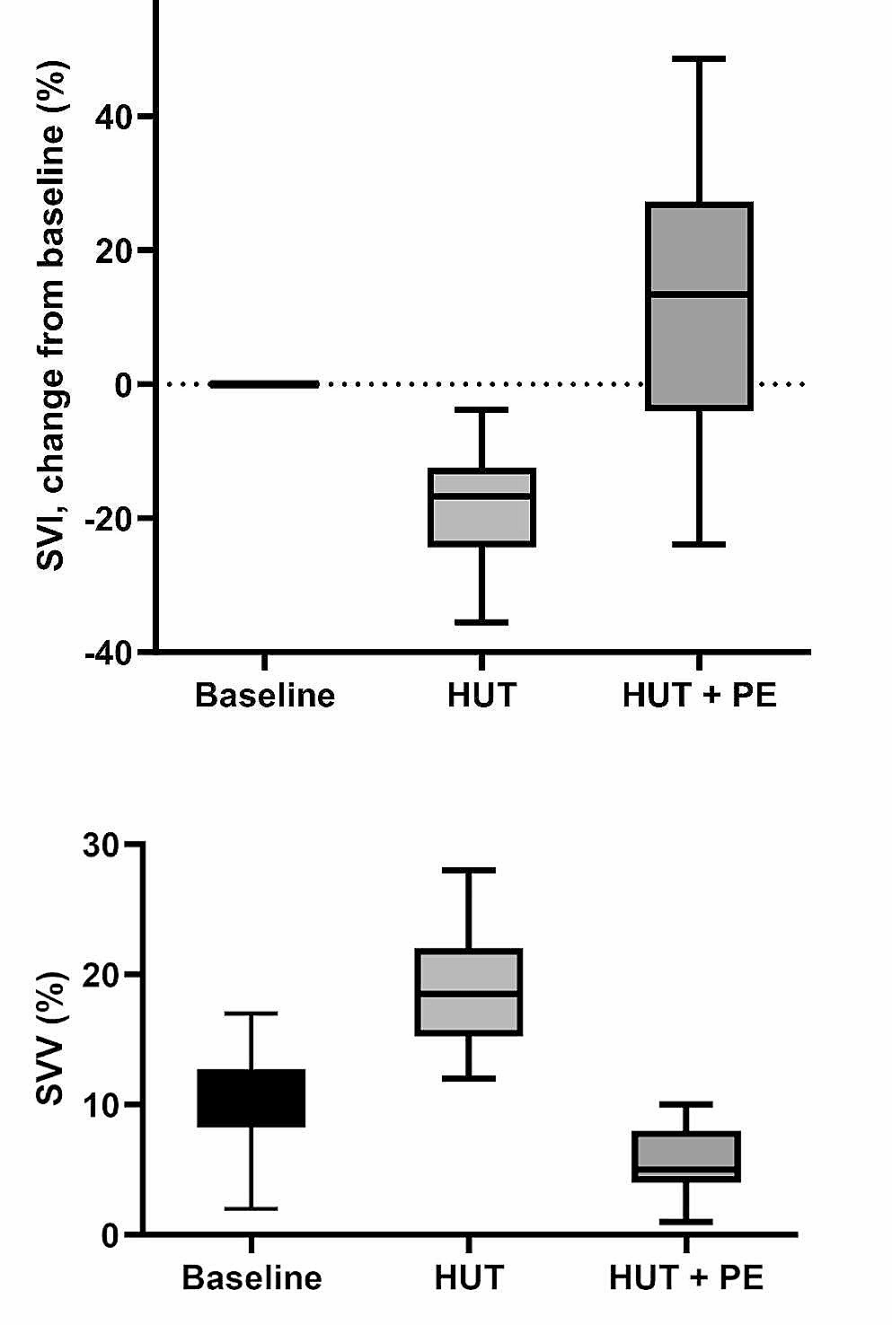
Effect of head-up tilt and phenylephrine infusion on stroke volume index and stroke volume variation Relative changes of Stroke Volume Index (SVI) and absolute values of Stroke volume Variation at baseline, during Head-Up Tilt (HUT) and during Head-Up Tilt with Phenylephrine infusion (HUT + PE). All differences: *p* < 0,001

With PE-infusion during HUT position CI increased by 18% to 2,6 ± 0,5 L/min*m^2^ (Fig. 1), reaching baseline values. MAP increased to 84 ± 14 mmHg. During HUT SVI decreased by 19% from 43 ± 7 mL to 35 ± 7 mL while SVV increased to 19 ± 4%. All patients had a SVV > 12% during HUT and were thus preload dependent. With PE-infusion in HUT position SV increased by 40% to 49 ± 11 mL and SVV decreased to 6 ± 3% (Fig. 2), with all patients having SVV < 12% as specified in the experimental protocol. All values *p* < 0,001. Heart rate was stable from baseline to HUT position but decreased from 63 ± 8 to 53 ± 8 with PE-infusion (*p* < 0,001).

**Table 2:** Effect of head-up tilt and phenylephrine infusion on cardiovascular variables

	SVI (mL/m^2^)	CI (L/min*m^2^)	MAP (mmHg)	HR	SVV (%)
Baseline	43 ± 7	2,6 ± 0,4	64 ± 10	62 ± 9	10 ± 3
HUT	35 ± 7	2,2 ± 0,4	43 ± 8	63 ± 8	19 ± 4
HUT + Phenylephrine	49 ± 11	2,6 ± 0,5	84 ± 14	53 ± 8	6 ± 3

All values mean(± st.dev.)

HUT = Head-Up Tilt; SVI = Stroke Volume Index; CI = Cardiac Index; MAP = Mean Arterial Pressure; HR = Heart Rate; SVV = Stroke Volume Variation

Statistics: All HUT values (except HR) different from baseline values, *p* < 0,001. All HUT + Phenylephrine values different from HUT values, *p* < 0,001

## Discussion

This study using indicator dilution technique is the first of its kind to demonstrate a clinically relevant augmentation of cardiac output using a pure vasoconstrictor in preload dependent surgical patients (Fig. 1). This supports the concept of using vasopressor preload modulation to increase venous return and CO in preload dependent patients.

Hypotension is common during general anaesthesia and so is administration of vasopressor. Traditionally therapeutic vasoconstriction has been thought to decrease cardiac output by increasing systemic blood pressures and thus afterload [13]. Besides PE norepinephrine (NE) is an extensively used vasopressor. As opposed to PE, NE also has some β-adrenergic effect potentially mitigating the effects of the increased afterload; whereas PE, with pure α1 stimulation supposedly decreases CO [4]. Indeed, some argue that phenylephrine should be avoided due to its proposed detrimental effect on CO [14]. On the other hand, some propose the more nuanced view that vasopressor may indeed increase CO if the heart is operating on the ascending part of the Frank-Starling curve: With CO limited by insufficient venous return, i.e. preload dependency [5, 15]. To this date the augmentation of CO by vasopressor preload modulation has not been demonstrated using a technique indifferent to alterations of vascular tone, such as indicator dilution technique. We believe that the lack of methodologically robust evidence for increasing CO by venoconstriction may be partly responsible for these discrepancies of opinion. Thus, PE provides the “purest” drug for pharmacological venoconstriction, and any increase in CO by PE-infusion must be due to increased venous return by recruitment of unstressed volume. As we wanted to describe only the effects of venoconstriction during conditions of decreased venous return, not confounded by inotropic effects, we chose PE for this study.

The LiDCO Unity monitor can measure CO using lithium dilution technique. This method has a high signal-to-noise ratio as lithium does not naturally occur in plasma. A minimal first pass loss from plasma supports precision and rapid redistribution makes repeated measurements feasible [11, 16]. Lithium dilution has been shown to have a very high precision, i.e. reproducibility [17].

Indicator dilution CO-monitoring is based on the Stewart-Hamilton principle that blood flow can be determined from the rate of change in the concentration of a substance added to the blood stream. These methods are notably not affected by alterations of vascular tone. Thermodilution using pulmonary artery catheter is the classical gold-standard, but newer less invasive methods such as Lithium dilution, show comparable, or better, precision – even in patients with varying cardiac outputs [11, 16–18].

General anaesthesia is associated with decreased sympathetic outflow, reducing vasomotor control of circulation [19–21]. Thus, head-up tilt during GA will often lead to preload dependency due to reduced venous return by gravitational pooling. This was also the case in this study where all patients became preload dependent with HUT as assessed by stroke volume variation (SVV). Stroke volume variation is a reliable functional parameter used to assess preload dependency. With general anaesthesia and stable heart rate the only variation seen in SV is induced by positive pressure ventilation affecting preload [22, 23]. Thus, with preload dependency the heart will be operating on the left steep part of the Starling curve being susceptible to marked alterations of preload with positive pressure ventilation; with increasing preload the heart will operate on the right, flatter part of the curve with less influence by ventilation – i.e. SVV corresponds to the slope of the Starling curve. A SVV higher than 9–13% will mean that the patient is preload dependent, and thus have a significant increase in SV/CO with volume expansion [23]. In this study we used SVV to verify that: (1) The patients were preload dependent with HUT, and (2) That PE-infusion during HUT did in fact recruit preload and abolish preload dependency (Fig. 2).

Cardiac output – flow through the circulatory system – is intimately related to venous return to the right heart [15, 24]. Venous return flow is dependent on the pressure gradient from venous side of the circulation towards the right atrium. This gradient can be modified by increasing stressed volume by administering vasopressor promoting venoconstriction [5]. As the pressure gradient towards the right atrium increases so does SV and CO until cardiac function is limited by the cardiac ventricles ability to accept a higher filling pressure/- volume, i.e. the flat part of the Starling curve. Thus, CO is always a balance between venous return and cardiac function. Whenever venous return is the limiting factor during positive pressure ventilation SVV will be high. We did not directly monitor venous return gradient, but the marked decrease in SVV with PE-infusion illustrates how stressed volume was increased, shifting the circulation from being limited by venous return to being limited by the cardiac function curve. This corresponds to the documented increase in CO with increased venous return (and decreasing SVV) until cardiac function limits further increase in flow, i.e. abolished preload dependency.

With PE-infusion SVV decreased to 6 ± 3% demonstrating that the heart was again operating on the flatter part of the Starling curve. This augmentation of preload – vasopressor preload modulation – led to a 40% increase in SVI (Table 2; Fig. 2; *p* < 0,0001). CI increased by 18% (Fig. 1) returning to baseline values (*p* < 0,001). With PE-infusion we observed relative bradycardia in our patients (Table 2). This reflex bradycardia is well known, especially in obstetrics, as PE-infusion is a well-established prophylactic treatment for spinal anaesthesia induced hypotension during cesarean section. As implied in the term “reflex” it is thought to arise from activation of baroreceptors in the carotid sinus [25], and not from a direct action of PE itself. This is also a plausible mechanism in our patients as MAP with PE-infusion was substantially higher than at baseline (84 vs. 64 mmHg; Table 2). However, even with increased afterload and reflex bradycardia, vasopressor preload modulation still increased CI.

We used head-up tilt during the sympatholysis induced by general anaesthesia as a model for preload dependency. This may not exactly reflect the physiology of preload dependency due to other causes, i.e. reduced circulating volume. Most likely the effect on the heart is the same in preload dependency of either cause: Venous vasoconstriction increases preload and thus CO. However, a severely reduced circulating volume will lead to tissue hypoperfusion, even with preload restored by (excessive) vasoconstriction. Conversely: The “classical” approach to preload dependency in the perioperative setting is to administer fluids to abolish preload dependency [3, 23] – even though preload dependency during GA may often be attributed to vasoplegia rather than hypovolaemia. As such, this approach may lead to excessive fluid administration associated with multiple complications [26, 27]. Thus, future research should focus on developing the concept of “safe vasoconstriction”, i.e. when is it safe to use vasopressor preload modulation for preload dependency, and when should volume expansion be used [28].

## Conclusion

This study using indicator dilution technique is the first of its kind and underpins the concept of vasopressor preload modulation to increase CO in preload dependent patients. We demonstrated an 18% increase in CI with infusion of a pure α1-adrenerg vasopressor during preload dependency induced by head-up tilt. This is the first human trial describing the physiology of this common clinical intervention with a methodology unaffected by alterations of vascular tone.

## Data Availability

No datasets were generated or analysed during the current study.
